# Acute mania in patient under tamoxifen

**DOI:** 10.1192/j.eurpsy.2021.1155

**Published:** 2021-08-13

**Authors:** H. Ghabi, M. Karoui, S. Ben Salem, R. Kamoun, F. Ellouz

**Affiliations:** 1 Department Of Psychiatry G, Razi hospital, Manouba, Tunisia; 2 Department Of Psychiatry G, razi hospital, manouba, Tunisia; 3 Department Of Psychiatry G, Razi hospital, manouba, Tunisia

**Keywords:** tamoxifen, bipolar disorder, protein kinase C, manía

## Abstract

**Introduction:**

Tamoxifen is an antioestrogen agent used in breast cancer treatment. According to some guidelines, this molecular was also proposed for the treatment of acute mania. In fact, Tamoxifen inhibits the intracellular action of the protein kinase C (PKC), which is the direct target in the treatment of mania episodes. Lithium and valproate have also the same action.

**Objectives:**

We aimed to show the case of an acute mania under an inhibitor PKC treatment and insisted that other studies are recommended.

**Methods:**

Case report description and research on medline, pubmed with the keywords: Tamoxifen, Bipolar disorder, protein kinase C,mania.

**Results:**

We reported a case of a 53-year-old woman with past history of unipolar depression. In 2018 when she was diagnosed with breast cancer. She received antidepressant drugs but she interrupted the treatment after a few months. She was treated for her breast cancer with mastectomy, radiotherapy, and 20 mg per day of Tamoxifen prescribed since Mars 2018. She had been admitted in June 2019 in our department for acute mania. The patient received Tamoxifen as it was prescribed. She was not taking any concomitant medications. No history of drug abuse was reported. Medical examination, laboratory, and radiological investigations did not indicate any medical pathology.

**Conclusions:**

In our case, Tamoxifen had not ovoid the acute mania in spite of its Known anti-manic properties as reported in the literature. Possible neurobiological effect of tamoxifen on the nervous system should be studied to evaluate the safety of this treatment mainly in patients with bipolar disorder.

